# FIGURING OUT LIFE AFTER COVID-19: A QUALITATIVE STUDY FROM SWEDEN

**DOI:** 10.2340/jrm.v55.11931

**Published:** 2023-12-11

**Authors:** Alexandra C. LARSSON, Karin TÖRNBOM, Katharina S. SUNNERHAGEN, Annie PALSTAM, Hanna C. PERSSON

**Affiliations:** 1 Department of Clinical Neuroscience, Institute of Neuroscience and Physiology, Sahlgrenska Academy, University of Gothenburg; 2 Department of Occupational Therapy and Physical Therapy, Sahlgrenska University Hospital; 3 Department of Social Work, University of Gothenburg; 4 Department of Public Health and Community Medicine, Institute of Medicine, Sahlgrenska Academy, University of Gothenburg; 5 Department of Neuroscience, Sahlgrenska University Hospital, Gothenburg; 6 School of Health and Welfare, Dalarna University, Falun, Sweden

**Keywords:** COVID-19, long COVID, rehabilitation, qualitative research, inpatients, recovery, activities of daily living, follow-up studies, health literacy, return to work

## Abstract

**Objective:**

To obtain a deeper understanding of the daily life experiences of working aged people during the year following hospitalization due to SARS-CoV-2 (COVID-19), with a focus on functioning in daily life and return to work.

**Design:**

An explorative qualitative study using individual interviews.

**Subjects:**

A purposive sample was selected of persons who had received inpatient hospital care, had been discharged approximately 1 year previously and were of working age.

**Methods:**

Semi-structured interviews were conducted with 11 participants (9 men, 2 women). The interviews were transcribed and analysed with inductive thematic analysis.

**Results:**

Four themes were identified. Navigating health, with or without support from healthcare, was described as challenging when managing consequences of COVID-19. Participants struggled with a lack of energy that interfered with daily life. It was a trial-and-error process trying to use familiar strategies in new ways to manage. The return to work process was facilitated by own strategies and support.

**Conclusion:**

This study contributes increased knowledge of everyday life experiences of people 1 year following hospitalization due to COVID-19. The lack of energy and a struggle to manage health while navigating the healthcare system emphasize the importance of strengthening personal and organizational health literacy to facilitate the recovery process after severe COVID-19.

LAY ABSTRACT

This study interviewed 11 participants (9 men and 2 women) about their daily life 1 year after being treated at hospital due to severe SARS-CoV-2 (COVID-19) infection. The interviews were transcribed and analysed qualitatively. Four main themes were identified. Participants described that it was challenging to navigate the healthcare system when managing the consequences of COVID-19. Participants also described struggling with a lack of energy that interfered with daily life. Furthermore, they described that previous experiences and strategies were used as a way to move forward. Returning to work was facilitated by own strategies and by support. This study contributes increased knowledge about daily life experiences 1 year after severe COVID-19. Lack of energy and a struggle to manage health while navigating the healthcare system stress the importance of strengthening accessibility of healthcare services as well as the patients’ competency in looking after their own health in order to facilitate the recovery process.

Common symptoms of severe acute SARS-CoV-2 (COVID-19) disease include dyspnoea, fever and cough (1, 2), which might require hospitalization (1). Recovery after severe COVID-19, 6-months after hospital discharge has been described as a challenging process, with patients being concerned about ever being fully recovered (3). Survivors have described struggling with long-term consequences of COVID-19 (4), and it remains unknown how COVID-19 will affect functioning over time. Long-term consequences can be, but are not limited to, fatigue, headache, attention difficulties and symptoms of anxiety and depression (5, 6). While studies indicate that long-term consequences of COVID-19 improve over the first year, some symptoms, such as shortness of breath, anxiety and depression may increase (5). Early in the pandemic, it was argued that rehabilitation should play an important role in the sub-acute phase of COVID-19 (7, 8). Currently, over 3 years after the initial outbreak of COVID-19, there is still a need for rehabilitation in some people who fell ill during the first waves of the pandemic, although rehabilitation needs may have decreased with milder versions of the virus in later pandemic waves. Reduced physical functioning 1 year after hospitalization has been reported in patients with severe COVID-19, as well as experiences of a slow and difficult recovery and a challenging rehabilitation process due to limited clinical support and sometimes contradictory advice (9).

For people of working age, a COVID-19 infection often involves having to take sick-leave, sometimes for several months in people who have had more severe illness and thus received in-hospital care (10). Furthermore, the ability to return to work after long-term sick-leave due to severe COVID-19 infection has been described as a sign of a successful recovery (11). Return-to-work is of importance for daily life as it provides a social context and a feeling of getting back to life again, as opposed to being on sick-leave, which has been described as stressful (11).

While some data are emerging on recovery over time after the infection (5, 9, 12), it is not yet fully understood how previously hospitalized patients have experienced the year of recovery after COVID-19. Being hospitalized with severe COVID-19 could be seen as a proxy of the initial severity of COVID-19 (13). In addition, questions remain about how people experience their rehabilitation, overall recovery and everyday life over a long period of time after severe COVID-19 (3, 9).

A qualitative approach may be useful for capturing the experience of recovery after COVID-19 in order to understand how individuals after a severe infection coped in the aftermath of COVID-19. The aim of this study was to obtain a deeper understanding of the daily life experiences of working age people during the year following hospitalization due to COVID-19, with a focus on functioning in daily life and return to work.

## METHODS

### Study design

This was a qualitative interview study with an exploratory approach. Individual interviews were performed with participants previously hospitalized for COVID-19, 1 year after discharge. The interviews were analysed using thematic analysis (14). Two patient partners were involved in the data collection and analytical process, by sharing their experiences during the development and pilot testing of the interview guide and thereafter validating the analysis through discussions of preliminary results.

### Ethics considerations

This study was approved by the Swedish Ethical Review Authority (Dnr: 2020-03046, 2020-03922, 2021-00444, 2021-03556) and followed the principles of the Declaration of Helsinki. All participants signed written informed consent prior to the interviews. The study followed the consolidated criteria for reporting qualitative research (COREQ) guidelines (15).

### Participants

Purposive sampling was applied to recruit individuals who had been included in the “Life in the time of COVID study in Gothenburg” (GOT-LOCO). The GOT-LOCO cohort comprised individuals who were hospitalized due to COVID-19 (July 2020 – February 2021 (first and second wave of the pandemic) in the Västra Götaland region, Sweden. The inclusion criteria were: patients hospitalized due to COVID-19, non-contagious at the time of inclusion, hospital care ≥ 5 days, ≥ 18 years old and community dwelling prior to hospitalization (16). Patients were excluded if they were unable to provide informed consent, had a life expectancy of less than 12 months, or were not Swedish residents.

To capture the current aim, participants were purposively selected to generate a heterogeneous sample from the GOT-LOCO cohort, based on the following criteria: approximately 1 year after hospital discharge, people of varying age within the category “working age” (18–65 years old), both men and women, different levels of education, diversity in hospital care including length of stay and diversity of symptoms reported at the 3-month follow-up (17). Fourteen persons were contacted by an invitation letter followed by a phone call. Also, further recruitment of participants was planned if needed. In total, 12 persons agreed to participate, among whom 1 woman later declined. The final sample consisted of 9 men and 2 women with a median age of 57 (range 43–63) years at the time of the interview (see Table I). At the time of the interview, 4 subjects were working full-time, 3 were on full-time sick-leave, and 4 had part-time sick-leave.

**Table I:** Participants’ characteristics

Characteristics (total *n*=11)	
Men, *n*	9
Age, median (range)	56 (45–62)
Education, *n*	
High-school	5
University	6
Work prior COVID-infection, *n*	
Employed	7
Self-employed	3
Unemployed	1
Length of stay in hospital, median (range)	19 (6–178)
Treated in ICU, *n*	7
Length of stay in ICU, median (range)	14 (7–85)
Treated in specialized in-patient rehabilitation unit, *n*	3
Received in-patient information regarding further rehabilitation in primary care, *n*	9

### Data collection

Individual semi-structured interviews with open-ended questions were conducted by the second author (KT), who had no previous relation to the participants. KT is a woman with a PhD in medicine and is a social scientist with long-term experience in qualitative methodology. Nothing regarding the interviewer’s personal interests, potential preconceptions or thoughts on the subject was conveyed to the participants, either before, during, or after the interview. Information about KT’s educational and professional background was communicated to the participants at the time of the interview. Upon request, 5 interviews were conducted by phone, 4 through videocall/digital platform and 2 were conducted face to face. The interviews were conducted using a semi-structured interview guide (Appendix S1) with open-ended questions that had been revised from the original pilot tested interview guide after discussions among all authors and 2 patient partners. The interview started with a few opening questions and continued with open-ended questions that were focused on the purpose of the study. The interviews were held in February 2022 and had a duration of 33–73 min, median 43 min. After each interview, KT made written notes with reflections about the content. All interviews were conducted in Swedish, were audio-recorded and transcribed verbatim. No further data collection was deemed to be necessary, given that the material was rich and nuanced, and the research questions were answered satisfactorily. The data collection generated an extensive material, which, in the analytical process, was separated into 2 articles, the current study and a previous study. The previous study of life experiences at 1 year after hospitalization for COVID-19 in Sweden focussed on patients’ mental health and social and emotional processes (18).

### Data analysis

To capture the participants’ experience without any predetermined theoretical framework, the interviews were analysed using inductive thematic analysis according to Braun and Clarke (14) (Table II).

**Table II:** Steps of thematic analysis according to Braun and Clarke

Steps	Description of steps
1	Getting familiarized with the data; verbatim transcribing, reading, and re-reading, taking notes for ideas.
2	Generating primary codes; coding interesting features of the data-set
3	Exploring the data-set for themes; grouping codes into possible themes
4	Revision of the themes; double checking if the themes work in relation to the codes and the whole data-set
5	Defining and naming the themes; continuing the analysis to refine the specifics of each theme
6	Generating the report; finalizing the analysis, selecting vivid extracts of meaning units reflecting the analysis and leading back to the research question, writing up the report.

Analysis of the data was mainly carried out by the first and second authors (KT and AL). AL is a female physical therapist and a PhD student with no previous experience in qualitative research who initially enrolled the patients into the GOT-LOCO study. AP supported and supervised the analytical process. AP is a female physical therapist with a PhD and long-term experience in qualitative research. The first step in the analysis was to become familiar with the entire material (14). Thus, all interviews were thoroughly listened to and were read and re-read separately by the authors (AL, KT). In the second phase, codes relevant for the research questions were colour-marked separately by the authors (AL, KT). In the third step, discussions were held to identify themes and potential sub-themes, albeit no sub-themes were used in this study. The identified themes were then revised in step 4, checking to make sure that the themes matched the codes and the dataset as a whole. This was carried out by AL, KT and AP. To ensure validity of the themes in relation to the data content, the process of analysis continuously shifted between the entire material and the different parts of the data-set. In step 5, the analysis process was continued in order to refine specifics of each identified theme. The themes were then discussed among all the authors (AL, KT, AP, KSS, HCP) until consensus was achieved. KSS is a female medical doctor with a PhD and extensive knowledge of both rehabilitation medicine and qualitative research. HCP is a female physical therapist with a PhD and knowledge of qualitative research; HCP is manager of the GOT-LOCO project. Participants were not involved in comments on transcripts or providing feedback on findings. However, the patient partners were invited to a meeting with the 2 authors most involved in the analysis process (AL and KT) where they were presented with the preliminary results and discussed the preliminary themes in relation to interpretations of participant quotes. The last step of the thematic analysis consisted of finalizing the analysis and generating a report. Meaning units and quotes reflecting the research question were selected (KT, AL, AP). The coding process is shown in Table III.

**Table III:** Coding tree

Themes	Potential themes	Coded for	Example of meaning units from interviews
It’s difficult to navigate one’s health in uncertain times.	Fear of getting infected again or worsening one’s condition by exercising	Feeling uncertain about exercising and risk for infection	“I figure that I will wait a while to get back to exercising, until this Omicron version settles down.” (Man; ID 5)
		Being afraid to exercise without supervision	“No, I haven’t practiced that so much now, I don’t dare, now that I don’t have that device [pulse oximeter].” (Man; ID 6)
	Lack of rehabilitation and healthcare follow-up	You have to navigate healthcare and follow-ups by yourself	“No, you’re on your own (concerning healthcare follow-up). It’s the same with rehabilitation. You had to figure it out for yourself. You’re healthy enough to go home, but then you don’t hear anything [no follow-up].” (Man; ID 5) “They say they’ll get back to you in 6 months, but it might as well be eight. I don’t think one should have to deal with these kinds of things.” (Man ID; 11)
Trying to lead life as usual, while lacking energy.	Experiencing a lack of energy	Being too tired to endure conflicts	“We lost the entire farm. I don’t think I would have ended up in this situation if only I had felt a little bit stronger, and if I had been my true self. […] Then, I would have been able to endure in a better way. […] But we didn’t speak up for ourselves, because I couldn’t take it.” (Woman; ID 10)
		Lack of energy	“I don’t know if it’s the energy or my commitment, I think both are lacking” (Man; ID 8)
	Can’t think clearly or make good decisions	Unable to think clearly and made irrational decisions	“I was very sad and upset. I shouldn’t have quit my job, right there and then. I should have contacted the Union, but my brain didn’t cooperate, and I couldn’t think, so I just resigned” (Woman; ID 9)
A trial-and-error process, using familiar strategies in new ways	Personality, previous experience influence choice of strategies	Recognizing small progress from previous experiences	“It’s progressing slowly 1 day at a time. There is a very small progression. Sometimes you feel like you’re on a plateau in your development, like when you work out a lot. I recognize that because I have been working out my whole life, so that…I recognize it” (Man; ID 5).
	Strategies used when starting to exercise	Exercising at a slower pace	“I started going to the gym. I started very slowly… There are elderly people taking classes. So, I started exercising with the oldies, they took things slowly and that suited me” (Man; ID 4)
	Strategies to cope in everyday life	Accomplishing fewer activities each day	“For example, if I’m going grocery shopping on a Monday, then I can’t take a shower or cook supper on that same day” (Woman; ID 9)
Wanting to be able to work again – strategies and support in the sick-leave process	Strategies to be able to work	Saving energy by working from home	“It was really good that one could work from home during most of this period (the pandemic). (…) When you’re sitting on a train (commuting to work), it is not demanding – no, but still, it is, because it’s not the same as being at home, resting…” (Man; ID 11)
		Strategies to save energy	“I try to work as much as I can during the first couple of hours, so I get it done, because in the afternoon I can’t think clearly” (Woman; ID 10)
	Managing one’s sick-leave	Support from colleagues	“It worked perfect (when I got back), they (colleagues) have adjusted my work tasks really well for my abilities… I could hardly walk when I got back.” (Man; ID 6)

## RESULTS

**Fig. 1:**
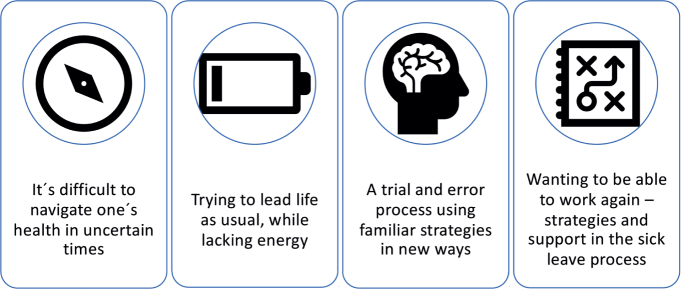
Illustration of the themes identified through the thematic analysis.

Most of the participants initially stated that they felt fine and had a good recovery after COVID-19. How-ever, as the interviews progressed, it became evident that they all had some remaining symptoms and that their everyday lives were, to various degrees, in one way or the other affected by these symptoms. Participants described trying to figure out life after COVID-19 and trying to find their way in this new reality, where 4 themes were identified (see Fig. 1).

### It is difficult to navigate one’s health in uncertain times

Participants perceived that information regarding rehabilitation services after COVID-19 was scarce or generally difficult to come by. When trying to navigate aspects related to their health status, participants described a feeling of being left to themselves. They described difficulties in getting in touch with healthcare facilities, and participants did not know how to go forward with making a healthcare contact when needed. Participants had experienced a huge contrast between the situation at the hospital where they had been strictly monitored and the situation at home, when they were left to manage their health by themselves. Participants described receiving sparse information about possibilities for further healthcare contacts when trying to contact primary healthcare for follow-up. They lacked information about whom to discuss their various symptoms with, or how to describe their difficulties, or when and if they would be followed up.

*It’s really hard, and above all it’s hard right now with post-COVID, because I know there is help to get, but I don’t get any help, because I don’t have enough of post-COVID (symptoms) as they say [laughs]. I have struggled and struggled [to get help], but I have given up now… I can’t keep hitting my head against the wall to get help.* (Woman; ID 9).

When they were unable to make healthcare contacts to find appropriate professional help, participants described trying to manage their symptoms and challenges on their own. This sometimes meant pushing their recovery processes forcefully with excessive exercise, which in some cases led to severe backlashes and to the rash conclusion that they could not exercise anymore. Others described fears about starting to exercise and were anxious that exercise could have a negative impact on their recovery process or worsen their condition. This led to under-activation among some participants.

*No, I haven’t practiced that so much, no. I don’t dare, now that I don’t have that device (pulse oximeter). So, I’ve been running a little bit, a few meters to catch the bus and so on. But afterwards I get really tired*. (Man; ID 6)

One participant emphasized that the cognitive screening before hospital discharge had raised an awareness of cognitive difficulties and contributed to the development of a way of coping with work and everyday life. The participants who had regular rehabilitation contacts during the year of recovery found this very useful. However, many were worried about being infected with COVID-19 again and therefore sometimes discontinued their rehabilitation contacts, in a sort of self-imposed lockdown.

*It was all up to me (the responsibility to continue with rehabilitation after leaving hospital). But even if the pandemic situation was different, I haven’t felt like going to public spaces or anything like that. I have continued to be cautious. In my opinion, it is common sense not to expose yourself if you don’t need to*. (Man; ID 3)

### Trying to lead life as usual, while lacking energy

Participants described that they were trying to maintain the life they had prior to COVID-19, despite having less energy than before, as a result of the infection. This caused irritation and frustration and was described as interfering with the participants’ daily life to a great extent. For example, usually enjoyable leisure activities were described by some as draining, leading to negative spirals and to more irritation and frustration. Participants expressed a need for more energy, although the best way to cope with this was not always clear, and many participants did not modify their activities or work tasks, but tried to carry on with activities as before. For example, participants had to put more effort into carrying out everyday tasks that had previously been done effortlessly.

Participants also described that they had no energy reserve, neither physical nor emotional. If anything happened outside the very ordinary, it could put them off balance and be very exhausting.

*Even the smallest thing, if added on top of my usual day […] makes me feel completely knocked out for hours. I am not responsive, I am completely off, I feel so tired. […] I usually sit on the couch in some sort of coma. I am not fully asleep, because I hear the sounds, but I cannot make sense out of the words. I don’t understand what my husband is saying. I can hear that he’s speaking, but I don’t understand what he’s saying.* (Woman; ID 9)

Participants described how their lack of energy and reduced capacities after COVID-19 sometimes meant severe consequences regarding important aspects of life. Participants described having acted in affect and having made irrational decisions in critical situations that had resulted in severe consequences for themselves and for their families. In some of these situations, participants described that they had realized too late what was about to happen and that they afterwards had regretted their irrational actions, since it had major life-changing consequences.

One woman described how she and her family had lost a legal dispute, even though they had the law on their side, due to her general lack of energy and mental confusion after COVID-19.

*We lost the entire farm. I don’t think I would have ended up in this situation if only I had felt a little bit stronger, and if I had been my true self. […] Then, I would have been able to endure in a better way. […] But we didn’t speak up for ourselves, because I couldn’t take it.* (Woman ID 10)

### A trial-and-error process, using familiar strategies in new ways

Participants tried to find ways to return to their former capacities. They described that they tried to apply strategies from previous experiences to this new context, which provided them with a framework for understanding and managing their ongoing recovery process.

*It’s progressing slowly one day at a time. There is a very small progression. Sometimes you feel like you’re on a plateau in your development, like when you work out a lot. I recognise that because I have been working out my whole life, so that… I recognize it.* (Man; ID 5)

While some found former coping strategies useful, others described feeling frustrated that their strategies did not seem to work to ease their remaining symptoms or to facilitate managing their daily life. Through a process of trial-and-error, participants described sometimes finding strategies to be effective, while in other cases, they described struggling to find useful strategies to manage daily life. Previous experiences of physical exercise, hospital care or rehabilitation were examples described by participants as being applicable when trying to move forward and recover after COVID-19.

*I have some coping tools from episodes of rehabilitation in my past, because I had experienced burn out […] and the tools from that time have helped me now too. […] Although the big difference is that when I was burnt out, I could exercise. The exercise was really good for my mental health as well. […] So, the big difference, and what bothers me the most, is that I cannot exercise now. Exercise has helped me so much before. […] I feel terribly sad that I can’t exercise*. (Woman; ID 9)

Further on, participants often experienced their positive attitude as a huge strength in allowing them to move forward and keep their spirits up. Being a positive person was seen as helping them not to dwell on the past, as well as helping with acceptance of one’s current life situation.

### Wanting to be able to work again: strategies and support in the sick-leave process

To participate in their own sick-leave process was described as an important strategy for return-to-work. A good communication with the physician was also perceived as very important, meaning that their experiences and opinions were heard and that they trusted their physicians’ recommendations regarding when they could return to work.

The opportunity to work from home was described as an important aspect contributing to return to work. Working from home meant not having to commute to work every day, which saved well-needed energy. Working from home also meant possibilities to take breaks when needed or to distribute work during the hours when they had the most energy, if possible. However, for some a pronounced lack of energy made return to work difficult even with flexible working conditions and opportunities to work from home. Some participants had a consistent strategy of trying to carry on as usual at work, even if it meant that they ended up completely exhausted on a daily basis.

*It was really good that one could work from home during most of this time period [the pandemic]. […] When you’re sitting on a train [commuting to work], it is not demanding – no, but still, it is, because it’s not the same as being at home, resting…* (Man; ID 11)

When returning to work, participants often found a boost to morale and confidence from supportive colleagues, as well as employers. Participants described how colleagues had been very understanding, showing support and sometimes releasing them from their heaviest work tasks, even several months after they had returned to the workplace. These acts of thoughtfulness made participants feel appreciated and grateful. The support of colleges and employers was, in some cases, described as an absolute prerequisite for being able to return to work at all.

*They [my colleagues] asked, like, “how are you coping?”, so they were responsive and asked if I was tired or if I couldn’t manage. […]. That shows that they appreciate me and that they appreciate my contribution and want me to stay. So, I mean, that definitely contributed to me wanting to be there as early as I could and to work full time.* (Man; ID 1)

## DISCUSSION

During the year that followed being hospitalized due to COVID-19, participants had tried to maintain their former ways of living with varying degrees of success. Feelings of exhaustion and lack of energy were especially troublesome, which supports previous findings of continuing symptoms and their consequences due to severe COVID-19 (3, 11, 19, 20).

In the theme “Trying to lead life as usual – while lacking energy” it was clear that participants struggled to lead life in the same way as they had before COVID-19, resulting in sudden feelings of exhaustion. The participants described a difficulty in knowing how to manage such sudden exhaustion and fatigue. While trying to live as before, the current participants felt challenged by their lack of energy and uncertain about how they should adapt to a more sustainable way of living, also shown previously (21). As described in a recent interview study, participants wanted to fight against fatigue to “manage the day”, and there was a strong desire to push one’s limits, even though the body and mind were not cooperating (22).

Returning to work was important for the participants to resume a normal day-to-day life, as described in the theme “Wanting to be able to work again – strategies and support in the sick-leave process”, which was also discussed in a previous study (11). In order to cope at work, participants expressed the importance of supportive employers and colleagues and their awareness of and willingness to adjust to situations related to the fatigue and other long-term symptoms after COVID-19. The need of support and having a constructive dialogue with employers and colleagues were highlighted as important during the return to work process in previous COVID-19 studies (11, 23, 24). During working life, participants were willing to push themselves and to give their all to successfully return to full-time employment, as described previously (11). In the return-to-work process, participants described having a dialogue with the primary care physician as key to making informed decisions on how to best proceed. Through this close communication, varying levels of health literacy among participants may have been acknowledged and addressed by the physician, facilitating successful strategies when returning to work. Furthermore, continuous support and rehabilitation for return to work, as well as patient involvement in the decision process, have previously been described as important for a well-functioning return-to-work process after severe COVID-19 (25).

The theme “A trial and error process using familiar strategies in new ways”, showed how participants tried to make their daily life manageable by using strategies that had been successful during previous life crises. However, these strategies were often experienced as useless when approaching the consequences of severe COVID-19. Furthermore, the theme “It’s difficult to navigate one’s health in uncertain times” showed that participants had not been able to establish rehabilitation contacts in order to find the right support to handle these difficult consequences of COVID-19.

Figuring out life 1-year post-COVID-19 was challenging, and it was evident that participants felt discouraged, frustrated and alone in their journey towards recovery. Based on these findings, primary care clinicians may need better knowledge and resources to deliver the care and support that patients with persisting consequences from COVID-19 may need (9). The current findings confirm previous results showing that participants experienced a lack of clinical support and contradictory advice regarding rehabilitation, 1 year after leaving hospital for COVID-19 (9). The main concern for the participants in the current study was about not being able to find the right rehabilitation services or the right healthcare facilities to contact during the first year after infection. Overall, participants found the healthcare system to be too complicated to comprehend and navigate. These findings indicate the importance of improving organizational health literacy within long-term rehabilitation for COVID-19 (26, 27). To meet the needs of patients with persisting symptoms post-COVID-19 puts great demands on organizational health literacy (28). Here, healthcare professionals are faced with a growing and heterogeneous patient group, seeking help and information based on different needs and life situations (27). Furthermore, the current results indicate the need for well communicated organizational health literacy when it comes to providing rehabilitation and advice on how to maintain sufficient levels of energy throughout the day, thereby enabling a more sustainable way of living. Better communication and a more individualized healthcare may facilitate better treatment and rehabilitation for these individuals based on their unique challenges, experienced symptoms and health consequences (28, 29).

Furthermore, considering the lack of energy described by participants, it might not be realistic to expect individuals with persisting consequences of COVID-19 to comprehend and manage their rehabilitation by themselves or to navigate a difficult healthcare system. In addition, previous research showed that a holistic and multifaceted rehabilitation approach is necessary when caring for persons with persisting symptoms post COVID-19 (22). The theme “A trial and error process using familiar strategies in new ways” showed that participants used coping strategies that had been successful when handling previous life crises or difficult experiences, not only in terms of rehabilitation, but also in terms of life in general, albeit sometimes they were inappropriate. For instance, not taking breaks when needed, but instead pushing forward and trying to fight fatigue was one way of dealing with difficulties that was counterproductive. This could also be related to varying levels of health literacy, in terms of the ability to make decisions that promote health in everyday life (28).

### Methodological considerations

The credibility of the results is supported by the purposeful selection of participants having been hospitalized due to COVID-19 and discharged approximately 1 year prior to the interviews, representing men and women of varying age, with different levels and length of hospital care and different levels of education (30). The interview material consisted of thoroughly articulated and nuanced answers and was therefore considered to provide a comprehensive understanding of the study aim. However, we consider the low number of women as a limitation when it comes to understanding health, rehabilitation, employment and daily life 1 year after COVID-19 from an in-depth female perspective. Furthermore, the relevance of the research question as well as the interview guide was supported by discussions with patient partners in an early phase of the study and when constructing and testing the interview guide as well as regarding the results. The confirmability (30) of the results was facilitated by the multi-professional group of researchers that brought different perspectives into the analytical process and through validation from patient partners in discussions of preliminary results. Finally, to facilitate transferability of the results (30), we have strived to provide careful descriptions of the involved researchers, the recruitment and selection process of the study sample and analytical process, as well as a careful selection of quotes to support the results. The transferability of the results must be judged by the reader. This study was performed in Sweden and the application of results to other contexts with potentially different healthcare systems needs consideration.

In conclusion, the findings of the current study contribute to increased knowledge about the everyday life experiences of people of working age, 1 year following hospitalization due to COVID-19. Lack of energy and a struggle to manage health while navigating the healthcare system stress the importance of strengthening personal and organizational health literacy to facilitate the recovery process after severe COVID-19.
